# Lipid Constituents of Diatoms (*Halamphora*) as Components for Production of Lipid Nanoparticles

**DOI:** 10.3390/pharmaceutics14061171

**Published:** 2022-05-30

**Authors:** Marta Marzec, Przemysław Dąbek, Andrzej Witkowski, Fernanda Monedeiro, Paweł Pomastowski, Bogusław Buszewski, Izabela Nowak

**Affiliations:** 1 Department of Applied Chemistry, Faculty of Chemistry, Adam Mickiewicz University, Uniwersytetu Poznańskiego 8, 61-614 Poznan, Poland; marta.dabrowska@amu.edu.pl; 2 Institute of Marine and Environmental Sciences, University of Szczecin, Mickiewicza 16a, 70-383 Szczecin, Poland; pdabek@usz.edu.pl (P.D.); andrzej.witkowski@usz.edu.pl (A.W.); 3 Centre for Modern Interdisciplinary Technologies, Nicolaus Copernicus University in Torun, Wileńska 4, 87-100 Torun, Poland; fmonedeiro@gmail.com (F.M.); p.pomastowski@umk.pl (P.P.); bbusz@chem.umk.pl (B.B.); 4 Department of Environmental Chemistry and Bioanalysis, Nicolaus Copernicus University in Torun, Gagarina 7, 87-100 Torun, Poland

**Keywords:** lipid nanoparticles, diatoms, *Halamphora*, microalgae oil, fatty acids, factorial design

## Abstract

Lipid nanocarriers smaller than 200 nm may be used as pharmaceutical/cosmetic raw materials as they are able to penetrate the skin. The nanostructured lipid carriers (NLCs) based on microalgae oil (*Schizochytrium*) and lipids extracted from diatoms (*Halamphora* cf. *salinicola* (strain SZCZM1454A)) were produced by the HSH (high shear homogenization) method. Fatty acid profile of crude oil from diatoms indicated the presence of palmitoleic, palmitic, stearic acid, oleic and myristic acids as the most common fatty acids in the strain investigated. The quantitative composition and the synthesis condition of NLC dispersions were optimized by using the full factorial designs. The physicochemical parameters of the obtained lipid nanocarriers were characterized by SEM, DSC and XRD measurements and the fraction with the optimum parameters (size below 200 nm, polydispersity index not exceeding 0.2 and zeta potential higher than +45 mV) was selected for further study. The positive charge of the obtained lipid nanoparticles is beneficial as permits electrostatic bonding with the negatively charged skin surface. As follows from stability tests, the NLCs obtained could be stored at room temperature.

## 1. Introduction

Diatoms (*Bacillariophyceae*) are photosynthetic unicellular eukaryotic organisms with cell walls composed of biogenic opal (silicon dioxide). Diatoms are ubiquitous in terrestrial and aquatic habitats and play an important role in global cycling of oxygen, carbon dioxide, nitrogen, phosphorus, and silica [1]. The total number of established species and genera exceeding 60,000 and 1200, respectively, makes diatoms one of the most species rich organismic groups on the planet [2]. No wonder that planktonic diatoms are estimated to account for more primary productivity than all the rainforests [3,4]. In addition to being primary drivers in the cycling of major nutrients globally, these photosynthetic microorganisms provide other essential ecological services in terms of sustainability of planktonic and benthic habitats. Their position at the base of the aquatic food chain results from the metabolic activity of their cells which make diatoms the food and energy source for numerous invertebrates, micro- and meiograzers, but also vertebrates, e.g., benthic fish [5]. This important role in the food chain is based on the fact that diatoms primary storage materials are triacylglycerols (TAGs, e.g., [6]) and carbohydrates (chrysolaminarin, e.g., [7]). TAGs occur in diatom cells as oil granules, commonly named lipid droplets. In addition to being food for aquatic grazers, diatomaceous lipids can have numerous applications as they can be easily converted into biodiesel [8] and many more substances, see the summary in [9]. The ability to store lipid droplets is unique to diatoms and a few other *Stramenopiles* (e.g., [10]). 

**Figure 1:**
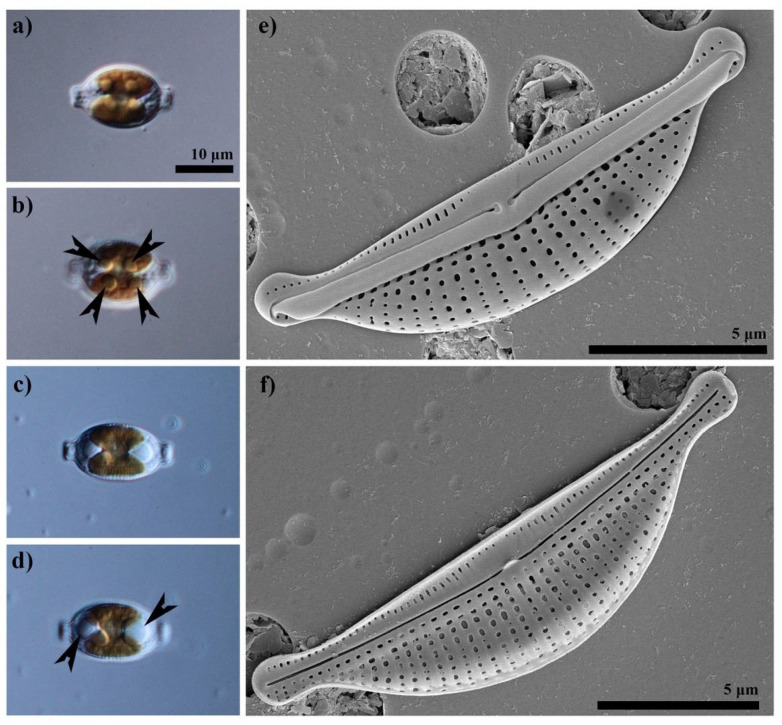
*Halamphora* cf. *salinicola* strain SZCZM1454A, isolated from Turkey. Live cells presenting chloroplasts arrangement (**a**,**c**) and lipids droplets (arrowed) accumulated in the cells (**b**,**d**). All scale bars are 10 μm. External (**e**) and internal (**f**) views of the siliceous frustule captured in SEM.

The potential of oil production is subject to significant changes across the diatom taxa and genera with some genera better than the others and some particularly rich in oil and called oleaginous forms. The latter group includes several taxa e.g., *Fistulifera solaris Mayama*, Matsumoto, Nemoto and Tanaka, 2014 [11]. Of more than 200 taxa characterized in the literature, a few diatom genera include numerous taxa of enhanced oil production potential [12]. Included in this group are, e.g., *Halamphora* (Cleve) Levkov [13] and *Nitzschia* Hassall (e.g., [14]), whose species are referred to as outstanding oil producers. It is estimated that the best diatomaceous strains may produce 40–70% of their body weight as oil [15]; however, numerous taxa produce only 10–20% of their body weight as oil [16,17]. A list of 25 diatom taxa for which oil yields have been assessed is given in [15]. Twelve of the taxa listed by the authors of this paper represent the genera of *Halamphora*, *Amphora* and *Nitzschia* and their lipid content ranged from 18 to over 45% of wet mass. For this study, we selected *Halamphora* cf. *salinicola* Levkov and Diaz (strain SZCZM1454A, Figure 1) isolated from an extreme environment of the hydrothermal spring in Turkey, as the fastest growing in high biomass and oil yield. The choice of diatoms from the *Halamphora* genus ensured high oil yield, high resistance and good growth in culture conditions. 

Nanostructured lipid carriers (NLCs) represent the second generation of lipid nanoparticles. The addition of a liquid lipid to the lipid matrix provided the possibility to increase the amount of incorporated active ingredient and improve the physical stability of the lipid nanoparticle dispersion [18,19]. For skin penetration (primarily transepidermal), lipid nanocarriers smaller than 200 nm may be used as pharmaceutical/cosmetic raw materials [20,21]. The use of lipids of natural origin in the production of lipid nanoparticles predisposes them to be used as ingredients with beneficial effects on the skin [22] or as carriers of drugs crossing the blood–brain barrier in the pharmaceutical industry. Microalgae oil (*Schizochytrium*) contains a system of polyunsaturated fatty acids—eicosapentaenoic acid (EPA) and docosahexaenoic acid (DHA)—which are responsible, among others, for protection of the skin against the negative influence of ultraviolet radiation, protecting it against photoaging and formation of discolorations. They also support the treatment of eczema and excessive skin roughness, ensuring the correct level of epidermal lipids [23,24].

The aim of the study presented in this paper was to produce nanostructured lipid carriers, enriched with lipids of natural origin, by applying a high shear homogenization (HSH) method and to optimize the composition of the resulting nanocarriers and synthesis conditions by applying the factorial designs. The reported study also involved the characterization of the physicochemical parameters of NLCs and the lipid matrix, and evaluation of their stability, which would allow verification of the potential for using optimized NLCs as pharmaceutical/cosmetic raw materials.

## 2. Materials and Methods

### 2.1. Diatom Isolation and Culture

Strain SZCZM1454A, morphologically identified as *Halamphora* cf. *salinicola*, has been isolated from the water sample originating from Köyceğiz Lake, Dalyan Channel in Turkey (36°50′34.4″ N, 28°37′54.6″ E), using micropipette technique under the inverted LM described in [25,26]. The diatom was grown in Petri dish enriched with artificial f/2 culture medium [27] with salinity adjusted to 35‰ and kept in a plant growth chamber (FITO1400i, Biogenet, Poland) with a 12:12-h light:dark photoperiod at 20 °C and illuminated with ca. 100 μmol photons m^−1^·s^−1^ of white light. The strain is stored at Szczecin Diatom Culture Collection (SZCZ), University of Szczecin, Institute of Marine and Environmental Sciences, Poland. For the lipids production, the strain SZCZM1454A was cultured in a 70 L cylindrical, air-lifted photobioreactor in the same conditions as specified above.

### 2.2. Crude Oil Extraction

Biomass was harvested every 30 days by means of free sedimentation (ca. 12 h) and centrifugation (2000 rpm/3 min), usually after the exponential growth phase, when the diatoms were accumulating lipid droplets in their cells. After collection, the biomass was dried at 70 °C for 24 h and powdered in a mortar. Extraction of crude oil was performed by the modified Folch method [28]. A portion of 1 g of the diatom powder was placed in a cellulose thimble into a Soxhlet device (FoodALYT RT 10, Omnilab, Bremen, Germany), together with a mixture of 30 mL of chloroform and 15 mL of methanol, both of purity ≥ 99% (Chempur, Piekary Śląskie, Poland). The extraction was carried out for 8 h bringing the solvents to boiling point. Then, the extract was transferred to a separation funnel, with the addition of 15 mL of distilled water, and a bottom chloroform phase, containing crude oil, was recovered to a glass vial and stored at 4 °C for further analysis.

### 2.3. Sample Preparation, Chemicals and Equipment Used in Fatty Acids Determination

The preparation protocol of a fatty acids sample was based on the procedure proposed in [29]. An aliquot of diatom crude oil (150 µL) was transferred to a 15-mL glass tube, together with 2 mL of methanol solution of sulfuric acid (1% (*v*/*v*) H_2_SO_4_ in methanol). The content was vortexed for 30 s. Next, the tube was capped and placed in a water bath at 60 °C, for 2 h, to promote the methylation of fatty acids. After this period, the sample was allowed to cool down to room temperature. Then, 2 mL of saturated sodium chloride solution (~6.14 M) and 1 mL of n-hexane were added. The content was vortexed for 40 s and the tube was set still for 2 min. Two visible layers were formed, and the organic layer (superior) was transferred to a clean 2 mL-centrifuge tube. A layer of anhydrous magnesium sulfate was added, and the tube was gently manually shaken. This step was taken to remove any excess of water from the recovered extract. The sample was centrifuged (2 min, 10,000 rpm) and the supernatant was transferred to a clean conical tube. The extract was dried under nitrogen 6.0 flow, at room temperature. The dried extract was resuspended in 100 µL of n-hexane. Finally, 1 µL of the sample was injected into the GC-MS system, in the splitless mode. The analyses were performed in triplicate (3 independent aliquots of obtained crude oil). 

Sulfuric acid 95.0–98.0% and sodium chloride ≥ 99% were obtained from Avantor (Gliwice, Poland). Magnesium sulfate anhydrous ≥ 99.5%, methanol and n-hexane (both HPLC grade) were purchased from Sigma-Aldrich (Steinheim, Germany). A VWR heating block and an Eppendorf^®^ 5424/5424R centrifuge were used during the sample preparation step.

### 2.4. GC-MS Analysis

The analyses were conducted on a 7820A gas chromatograph coupled with a 5977B GC/MSD and equipped with a G4513A autosampler (Agilent Technologies, Santa Clara, CA, USA). Inlet temperature was kept at 270 °C and carrier gas (helium 6.0) flow was set at 1.2 mL min^−1^. A HP-5MS capillary column (Agilent, Palo Alto, CA, USA) 30 m × 0.25 mm × 0.25 µm was used. The oven temperature program was as follows: initial temperature was 60 °C (held for 3 min), ramped to 280 °C at a rate of 6 °C min^−1^. The latter temperature was kept for 10 min, resulting in a run time of 49.70 min. Full scan spectra were acquired within a range of 35–700 *m*/*z*, at an electron ionization (EI) of 70 eV. A solvent delay of 4.5 min was applied. The quadrupole, ion source and transfer line were set to 150, 230 and 250 °C, respectively. Chromatographic data acquisition was performed using the MassHunter Workstation 10.0.368 (Agilent Technologies, Santa Clara, CA, USA). Peak areas were obtained through manual integration, in order to calculate relative compound response, which later was expressed in terms of the percentage of total fatty acid content. Compound identification was performed using the NIST17 mass spectra library. Each peak was searched manually, including baseline subtraction and averaging over a peak. A forward match quality of at least 750/1000 was applied as the lower match threshold. The actual EMV was 1490.9 V, using a gain factor of 4.99.

### 2.5. Lipid Screening

The procedure for the selection of solid lipid for the production of lipid nanoparticles was as follows: (i) appropriate amounts of selected solid and liquid lipids at a ratio of 2:1 were weighted; (ii) the components were heated to 10 degrees above the melting point of the solid lipid; (iii) the resulting mixtures were observed at 15 min, 30 min, 1 h, 24 h and 72 h after the system solidification. The aim of the experiment was to achieve the most homogeneous mixture possible resulting from solidification of the combination of the solid and liquid lipids tested.

In this work, four different solid lipids – Compritol^®^ 888 ATO (glyceryl dibehenate), Precirol^®^ ATO 5 (glyceryl distearate) from Gattefossé (Lyon, France) and Imwitor^®^ 900 K (glyceryl monostearate), Softisan^®^ 601 (a blend of glyceryl cocoate, glyceryl stearate and gliceryl ricinoleate) from IOI Oleo GmbH (Hamburg, Germany)—were selected for the screening approach. Miglyol^®^ 810 N (caprylic/capric triglycerides) from IOI Oleo GmbH, microalgae oil (*Schizochytrium*) from Norsan (Berlin, Germany) and *Halamphora* cf. *salinicola* (SZCZM1454A) crude oil (Szczecin Diatom Culture Collection, University of Szczecin, Poland) combined with microalgae oil (at different ratios: 1:1; 1:1.5; 1:2) were used as liquid lipids in the study. 

### 2.6. Production of Lipid Nanoparticles

Nanostructured lipid carriers were produced by applying a high shear homogenization method. The procedure involved the preparation of a lipid phase containing a solid lipid (selected during lipid screening—Imwitor^®^ 900 K), one of the liquid lipids, glycerol (Chempur, Piekary Śląskie, Poland) and hexadecyltrimethylammonium bromide (CTAB, used as a cationic surfactant) purchased from Chemat (Konin, Poland), and heated to 75 °C. Separately, an aqueous solution of the non-ionic surfactant Tween^®^ 80 was prepared and heated to 40 °C. Subsequently, a small amount of the aqueous phase was combined with the lipid phase and subjected to a two-step high shear homogenization (Ultra-Turrax^®^ DI 25 Basic, IKA-Werke GmbH, Staufen im Breisgau, Germany). In the next step, the resulting mixture was added to the rest of the aqueous solution of the surfactant under continuous stirring to a gradual cooling of the dispersion. The details of the method of production of the NLCs studied in this work is covered by a patent application in Poland—no. P.438767 and an European patent application—no. EP22460021 (Marzec M, Nowak I, Dąbek P, Witkowski A. A method of obtaining of lipid nanoparticles synthesised on the basis of marine microalgae (*Schizochytrium*) and lipids obtained from diatoms (*Halamphora*)).

### 2.7. Experimental Factorial Design

The quantitative composition of the NLC dispersions was optimized by using the 3^3^ factorial design. Three factors, each set at three concentration levels, were assumed as independent variables: (i) solid lipid content—Imwitor^®^ 900 K—4.0; 4.5; 5.0 wt.%; (ii) liquid lipid content—Miglyol^®^ 810 N, microalgae oil and *Halamphora* crude oil combined with microalgae oil—1.0; 1.5; 2.0 wt.%; (iii) non-ionic surfactant content—Tween^®^ 80—0.5; 1.0; 1.5 wt.%. Three physicochemical parameters were selected as dependent variables: (i) mean particle size (Z-Ave); (ii) polydispersity index (PDI) and (iii) zeta potential (ZP), which were determined with a Zetasizer Nano ZS (Malvern Instruments, UK). The 3^3^ factorial design required twenty-seven experiments for each of the liquid lipids tested (Table 1). The optimization was complemented by the selection of the speed of the two-stage high shear homogenization. In this part (3^2^ factorial design, Table 2), two factors, the speed of the first and the second homogenization stage, each fixed at three speed levels, were chosen as independent variables. The same parameters as those mentioned above in the 3^3^ factorial design were selected as dependent variables. The optimization of the synthesis condition was carried out for the selected samples containing each of the liquid lipids investigated. The statistical analysis involved an evaluation of measured data with the help of Statistica 10.0 software (Statsoft, Poland). The level of statistical significance was set at *p* < 0.05.

### 2.8. Physicochemical Characterization of Lipid Nanoparticles

Physicochemical parameters of the lipid nanoparticle dispersions—mean particle size, polydispersity index and zeta potential—were determined with a Zetasizer Nano ZS (Malvern Instruments, UK), using the techniques of dynamic light scattering (the light scattered by the sample at a backscatter angle of 173°) and electrophoretic light scattering [30]. The measurement was preceded by the preparation of aqueous solutions of the studied lipid nanoparticles (60 μL of the dispersion in 15 mL of distilled water), and the determination of the refractive index in each test sample using a Refracto 30 PX/GS refractometer (Mettler Toledo, Warsaw, Poland). The measurements were carried out at room temperature. The procedure was performed in triplicate for each test sample.

### 2.9. Characterization of Lipid Matrices

Characterization of the lipid matrices of NLCs was performed for the optimized samples by using scanning electron microscopy (SEM), differential scanning calorimetry (DSC) and X-ray diffraction (XRD). The morphology and shape of the lipid nanoparticles were established on the basis of images recorded using a FEI Quanta 250 FEG Scanning Electron Microscope (FEI Company, Hillsboro, OR, USA). Before the SEM analysis, the samples were prepared by drying the lipid nanoparticle dispersions at room temperature. The parameters of analysis are the magnification of 30,000× and the electron beam energy of 5 keV. The polymorphic forms of the lipid matrix of NLCs were evaluated on the basis of the results of thermal analysis performed with a DSC 8500 differential scanning calorimeter (PerkinElmer, Waltham, MA, USA). The procedure involved the filling of a 40-μL aluminum pan with a solid lipid sample or a sample of the dispersion of the lipid nanoparticles under study. The measurement procedure included the gradual heating of the sample from 25 °C to 90 °C in nitrogen flow (20 mL/min) at a scanning rate of 10 °C per minute, keeping the sample at 90 °C for 1 min, and then cooling it down to 25 °C at similar parameters. The results are presented in the form of DSC curves. Moreover, the crystallinity and polymorphic forms of the NLCs investigated were determined based on the results of diffraction analysis carried out with a D8 Advance powder diffractometer combined with a Johansson monochromator (Bruker, Billerica, MA, USA). To prepare the samples for analysis, the dispersions of lipid nanoparticles were dried at room temperature and the procedure was performed within the wide-angle range (2Θ = 6.0–60.0°).

### 2.10. Stability Study

The stability of model NLCs samples was evaluated using a Zetasizer Nano ZS instrument and on the basis of measurements of three characteristic physicochemical parameters: Z-Ave, PDI, and ZP. The stability test was performed 24 h after the synthesis of the dispersions of lipid nanoparticles (day 1) and after 30 days (day 30) by analyzing the samples stored at three temperatures (4, 25, and 40 °C).

## 3. Results and Discussion

### 3.1. Fatty Acid Profile of Halamphora cf. salinicola (SZCZM1454A) Crude Oil

**Figure 2:**
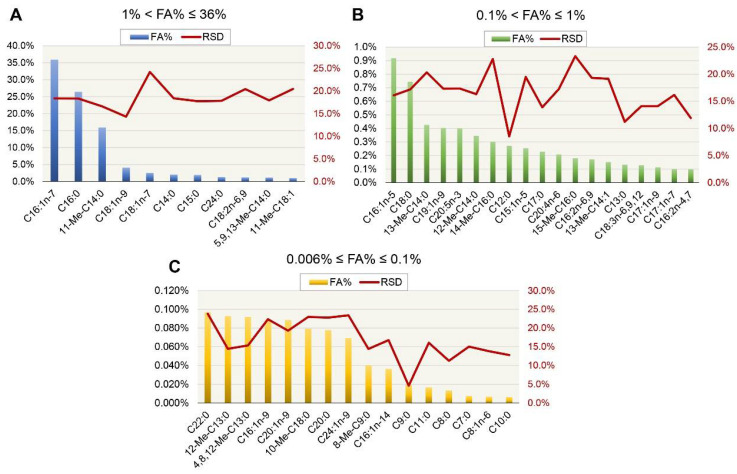
Fatty acid content in the crude oil diatom sample. The graphs show fractions of total fatty acids in the range of 1 to the maximum i.e., 36% (**A**), from 0.1 to 1% (**B**) and from 0.006 to 0.1% (**C**). FA = fatty acid; RSD = relative standard deviation; Me = methyl.

Based on the analysis of fatty acid methyl esters, a total of 46 different fatty acids were detected. From them, 34 (74% of the total) were straight chain fatty acids, whereas 12 (26%) were branched chain fatty acids. The examined diatom strain appeared to be able to produce fatty acids containing 7 to 24 carbon atoms. The number of saturated fatty acids (25, 54%) was slightly superior to that of unsaturated ones (21, 46%). In order to assess the method’s precision, the relative standard deviation (RSD) was calculated for each of the chromatographic peaks assigned to the compounds of interest. RSD ranged from 4.6 to 24.2%, obtained for nonanoic acid and 11-octadecenoic acid, respectively. 9-Hexadecenoic acid, hexadecenoic acid and 11-methyltetradecanoic acid were the species found in the highest proportions in the examined samples, of about 35.9, 26.4 and 15.9% of the total investigated fatty acid content, respectively. Figure 2 presents the average fractions of fatty acids verified in the crude oil sample. Figure 2A–C present the levels of fatty acids identified within the total content from 1 to 36%, 0.1 to 1% and below 0.1%. Table 3 presents detailed information on detected fatty acids and their respective relative percentage content in the sample.

The advantages of using diatoms as a lipids source is well recognized, these are especially of enormous species richness, with a number of lipid-rich taxa (in average accumulating 20%) and hence also a variety of fatty acids composition (e.g., for biodiesel production, food supplement, etc.). The relative ease in culture and as for some strains, the possibility of applying an environmental stress to enhance the amount of lipids produced [16,31,32]. In standard culture conditions *Halamphora* cf. *salinicola* strain SZCZM1454A accumulates ca. 22% of the lipids; however, due to nutrients deficiency (mainly phosphorus and silica). The amount of lipids in the cell may increase to about 40% (PD personal observation). It is well known that under nitrate deficiency, the carbohydrate contribution increased, while the protein contribution decreased. Inversely, phosphate deficiency increased the proportion of proteins and decreased carbohydrates contribution [33]. The most common fatty acids found in the strain studied, and also in the other diatoms [34], are as follows: palmitoleic, palmitic, stearic acid, oleic and myristic acids (Table 3).

### 3.2. Selection of Solid Lipids Compatible with the Liquid Lipids Tested

The solid lipid screening was performed to select the solid lipids compatible with the investigated liquid lipids—Miglyol^®^ 810 N, microalgae oil (*Schizochytrium*) and lipids extracted from diatoms *Halamphora* cf. *salinicola* (SZCZM1454A) combined with microalgae oil. Table 4 shows the results of the lipid screening carried out over time for the above set of solid lipids. As expected, the commercially available liquid lipid, Miglyol^®^ 810 N, formed a homogeneous mixture with most of the solid lipids tested after 72 h [35]. The exception was only Precirol^®^ ATO 5, which formed a heterogeneous mixture with Miglyol^®^ 810 N from the beginning of the experiment. For microalgae oil, two solid lipids (Imwitor^®^ 900 K and Compritol^®^ 888 ATO) were demonstrated to solubilize the liquid lipid over the time of 72 h. For *Halamphora* cf. *salinicola* crude oil, a satisfactory result was obtained only with Imwitor^®^ 900 K. Nevertheless, in order to standardize the synthesis and to objectively compare the results in the study, it was decided to choose Imwitor^®^ 900 K for the subsequent production of lipid nanoparticles, thanks to its lower melting point (≈60 °C) than that of Compritol^®^ 888 ATO (≈70 °C), which favors the application as pharmaceutical/cosmetic raw materials.

### 3.3. Optimization of the Quantitative Composition of Lipid Nanoparticles and the Conditions of Their Production

**Figure 3:**
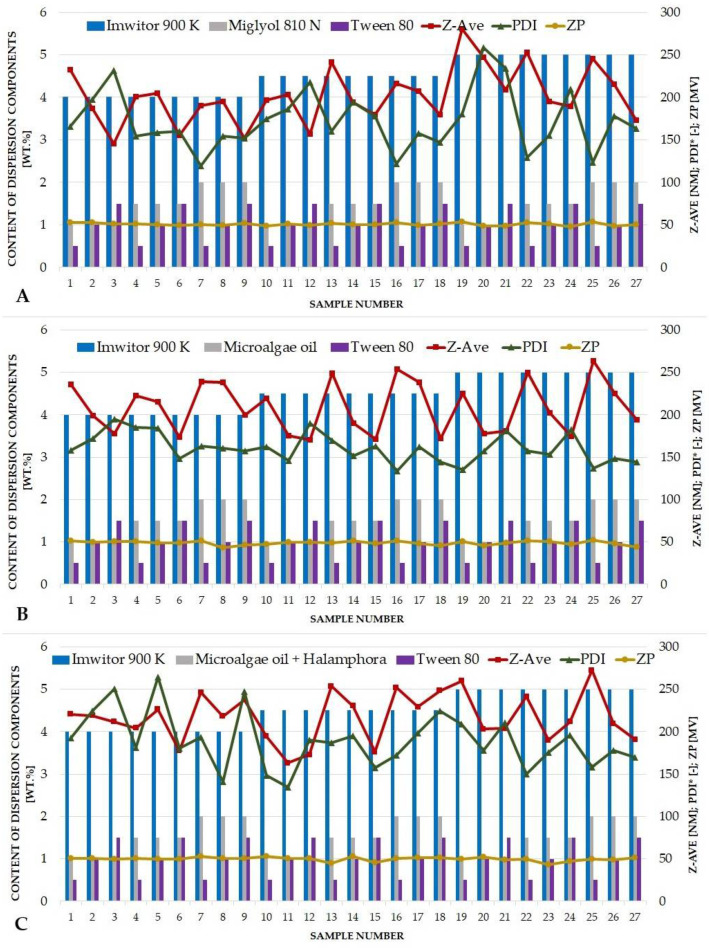
Results of 3^3^ factorial design for the NLCs containing Miglyol^®^ 810 N (**A**), microalgae oil (**B**) and a combination of microalgae oil and lipids extracted from diatoms *Halamphora* cf. *salinicola* (**C**) as the liquid lipid (PDI value multiplied by a constant value of 1000).

The experimental factorial design made it possible to establish the optimum quantitative composition and production conditions of the nanostructured lipid nanocarriers intended for the use as pharmaceutical/cosmetic raw material for skin penetration. Table 5 presents the detailed results of 3^3^ factorial design regarding the optimization of the contents of three ingredients of the lipid nanoparticle dispersions. For the NLCs containing the commercial liquid lipid—Miglyol^®^ 810 N (Figure 3A)—a dependence of the mean particle size on the amount of non-ionic surfactant was noted (*p* < 0.05). A decrease in the nanoparticles’ size with increasing Tween^®^ 80 content was observed, regardless of the amount of Miglyol^®^ 810 N. It was a characteristic of lipid nanoparticles based on non-ionic surfactants from the Tween^®^ group. These substances, especially Tween^®^ 80, make it possible to obtain nanolipid structures of relatively small particle size, and this effect deepens with increasing content of Tween^®^ 80 in the sample. Moreover, an increasing concentration of solid lipid caused a raise of the size of the nanocarriers (*p* < 0.05), which was also not an unexpected observation, since usually an increase in the content of solid lipid is associated with obtaining NLCs of larger size. Hence, it is up to the researchers, to find a compromise between a satisfactory solid lipid amount and the desired size of lipid nanoparticles. An inversely proportional relationship between PDI and Tween^®^ 80 content was noted for most of the samples (*p* < 0.05). It should be mentioned that Tweens^®^, in addition to their beneficial effect on the size of the NLCs, also make it possible to obtain dispersions with a high degree of homogeneity, without a tendency to agglomerate during storage. The value of zeta potential did not fluctuate significantly. The positive charge of the NLCs investigated is solely related to the presence of CTAB in the composition of the samples, and its content was fixed at a constant level during the experiments. For the NLCs containing microalgae oil (Figure 3B), the results were somewhat different. The contents of microalgae oil and Tween^®^ 80 had the greatest impact on the determined sample parameters (Z-ave, PDI). Their effect is particularly pronounced on the average particle size, as the value of this parameter increases with increasing amount the oil introduced (*p* < 0.05), while it decreases with increasing surfactant content (*p* < 0.05). The same tendency of changes in the mean particle size, that is its increase with the increasing oil content and the decrease with the increasing content of the surfactant, was observed for all samples, irrespective of the content of solid lipid. PDI did not follow such an unambiguous course, although it was notably dependent on the liquid lipid content (*p* < 0.05). The addition of microalgae oil, as a liquid lipid of natural origin, slightly disturbed the homogeneity of the lipid nanoparticles investigated, making the balance between the expected microalgae oil content and PDI value a key challenge for the authors of this study. The results for the NLCs with a combination of microalgae oil and *Halamphora* cf. *salinicola* (SZCZM1454A) crude oil were very similar to those obtained for the NLCs with microalgae oil only (Figure 3C). At first, we observed no effect of solid lipid content on any of the dependent variables. The main observation was a significant relationship between the contents of microalgae oil and Tween^®^ 80 and the mean particle size (*p* < 0.05). Surprisingly, the addition of another lipid of natural origin to the NLC composition did not disturb the tendency of changes in the mean particle size of NLC samples. The correctly selected method of nanostructured lipid carriers’ preparation with a special emphasis on the accurate dispersion of *Halamphora* cf. *salinicola* crude oil in the microalgae oil may have been crucial here. The trend of PDI changes was different. The zeta potential, on the other hand, remained mostly constant. The optimum composition of the designed NLC formulations was determined at the solid lipid:liquid lipid:non-ionic surfactant content ratio of 4.5:1.5:1.5 wt.% (sample no. 15) for each liquid lipid investigated. The composition of the samples chosen was a compromise between the maximum possible amount of solid and liquid lipid and the minimum of surfactant, at which the desired physicochemical parameters of lipid nanoparticles took the optimum values (particle size below 200 nm, PDI not exceeding 0.3, ZP higher than |±30 mV|). The positive charge of the obtained lipid nanocarriers is related to the presence of CTAB (a surfactant of cationic nature) in the composition of the dispersion and should be considered as a beneficial aspect in terms of the interaction between the negatively charged skin surface and the positively charged particles [36]. Compared to the results of other research groups [37,38], working in the area of similar dispersion components, our NLCs are characterized by a slightly larger particle size. Nevertheless, the PDI and ZP values obtained are very consistent. It should be remembered that our lipid carriers were obtained using a less demanding method, without applying high pressure or ultrasound, which has not been described before, for nanostructured lipid carriers based on lipids of natural origin.

**Figure 4:**
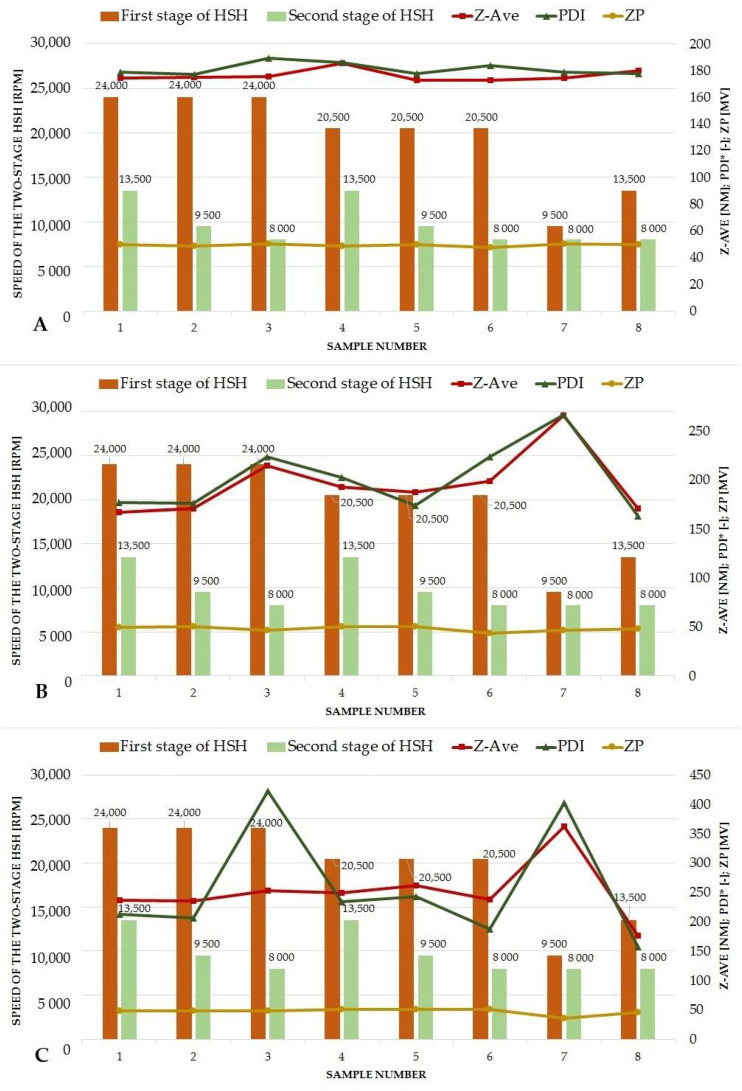
Results of 3^2^ factorial design for the NLCs containing Miglyol^®^ 810 N (**A**), microalgae oil (**B**) and a combination of microalgae oil and lipids extracted from diatoms *Halamphora* cf. *salinicola* (**C**) as the liquid lipid (PDI value multiplied by a constant value of 1000).

**Figure 5:**
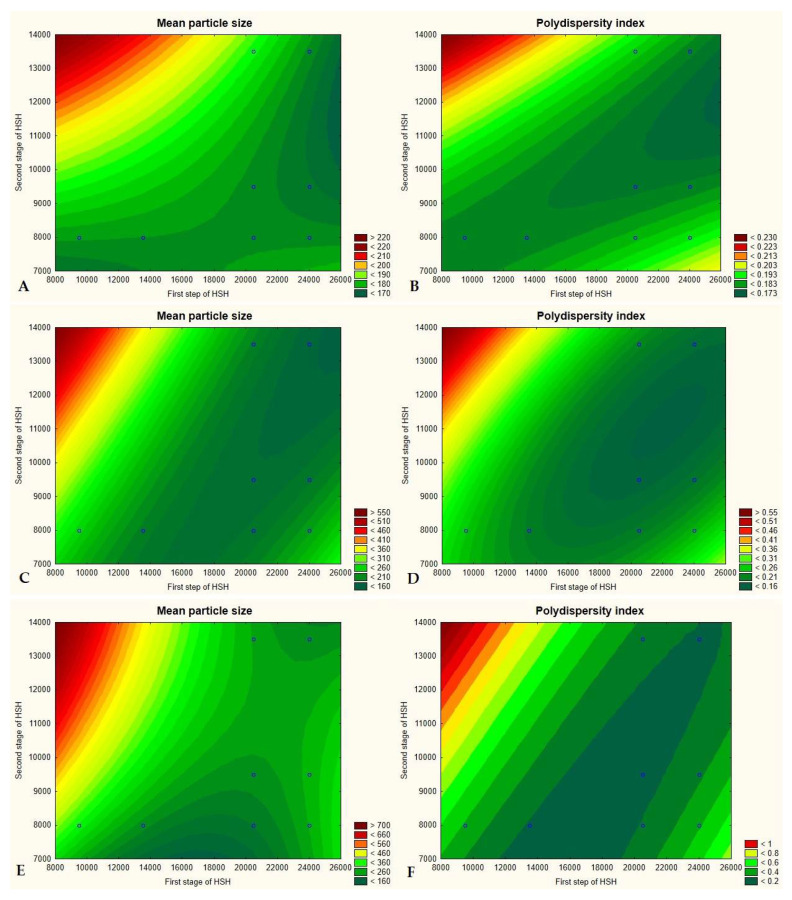
Response surface plots for the effect of the speed of two-stage homogenization on the mean particle size and polydispersity index of the NLCs containing Miglyol^®^ 810 N (**A**,**B**), microalgae oil (**C**,**D**) and a combination of microalgae oil and lipids extracted from diatoms *Halamphora* cf. *salinicola* (**E**,**F**) as the liquid lipid.

The second part of the optimization process concerned the conditions of the two-stage high shear homogenization, mainly the stirring speed. The results showed that a moderate speed of the first stage of homogenization and a much slower stirring speed at the second step were beneficial in terms of the response values of the experiments. Below the results obtained for individual samples will be discussed. At first. the factorial design was performed for the lipid nanoparticles enriched in Miglyol^®^ 810 N. No statistically significant changes in physicochemical parameters caused by modulation of the speed of each homogenization step were observed. It could be noted that the values remained comparable for all samples (Figure 4A). However, the response surface plots (Figure 5A,B) indicated that the most favorable option was a combination of a moderate speed of the first homogenization stage (13,500 rpm) and a very slow subsequent second stage (8000 rpm), at which the desired values of physicochemical parameters of lipid nanoparticles were maintained. Similar conclusions can be drawn for the NLCs containing microalgae oil. According to the results presented in Figure 4B, the sample with the optimum Z-ave and PDI was that obtained in the above-described way. Although the Anova analysis does not indicate statistically significant changes in the values of Z-Ave, PDI and ZP, the least favorable effect of the application of too fast (24,000 rpm) or too slow (9500 rpm) speed at the first stage combined with the minimum speed (8000 rpm) at the second stage of homogenization was observed (Figure 5C,D). It was identified as a cause of lower homogeneity of the lipid nanoparticle dispersions. The results obtained for the NLCs containing *Halamphora* cf. *salinicola* crude oil blended with microalgae oil were similar to those for the NLCs with the microalgae oil only (Figure 4C). These observations concerned in particular the polydispersity index. The use of the limiting velocities (24,000 and 9500 rpm) at the first stage of homogenization combined with the speed of 8000 rpm at the second stage had a significant negative effect on the PDI value (Figure 5F). However, also on the basis of this study the optimum sample proved to be sample 8, the same as indicated by the two above-discussed factorial designs (Figure 5E). The “model” lipid nanoparticle dispersions were homogenized with Ultra-Turrax at 13,500 rpm at the first stage followed by the speed of 8000 rpm at the second stage of homogenization. The choice was also determined by technological factors of the process, namely the combination of the HSH speeds reduced the foaming of the sample during the production of NLCs and permitted obtaining a homogeneous dispersion of lipid nanoparticles.

### 3.4. Analysis of the Results Characterizing the Lipid Matrices (SEM, DSC, XRD)

**Figure 6:**
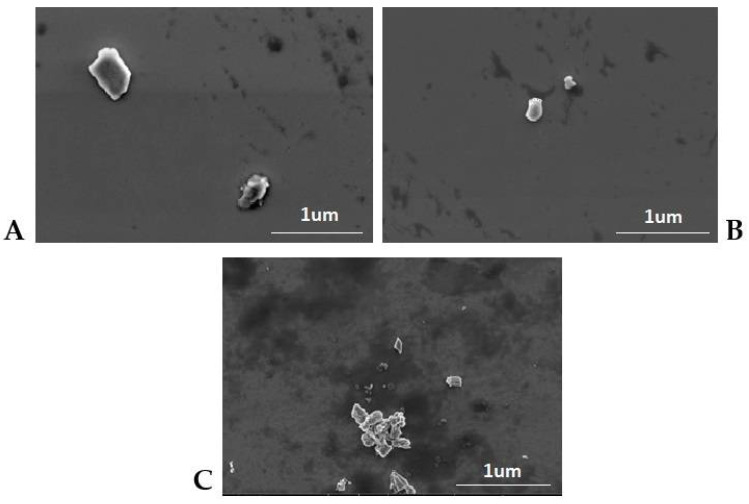
SEM images of NLCs containing Miglyol^®^ 810 N (**A**), microalgae oil (**B**) and combination of microalgae oil and lipids extracted from diatoms *Halamphora* cf. *salinicola* (**C**) as liquid lipid proved the irregular shape and the size in the range below 200 nm of the NLCs investigated.

**Figure 7:**
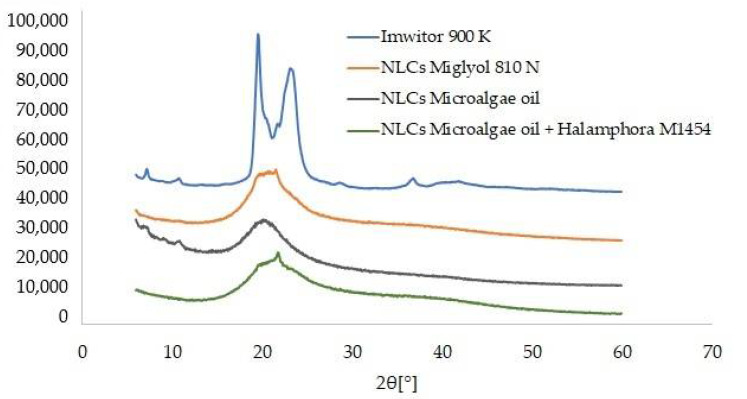
Comparison of diffractograms of the solid lipid and the studied dispersions of NLCs (the diffractograms are shifted by a constant value of 12,000 arbitrary units in relation to the previous diffractogram).

**Figure 8:**
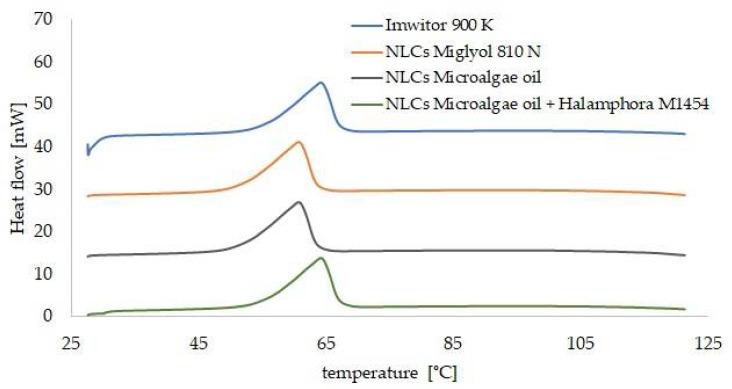
Comparison of DSC thermograms of the solid lipid and the studied dispersions of NLCs (the thermograms are shifted by a constant value of 15 mW in relation to the previous thermogram).

The samples selected as the optimum ones were further characterized by SEM, DSC and XRD methods. The SEM images (Figure 6) enabled evaluation of the shape and confirmation of the size of the NLCs investigated. For the second generation of lipid nanoparticles, i.e., the nanostructured lipid carriers, structures with irregular shape and uneven surface, are expected [39]. Such a morphology is caused by the presence of liquid lipid in the lipid matrix, whose addition affects the stability of the NLC dispersion. On the basis of the obtained images as well as DLS measurements, we may presume the presence of NLCs type II or III, i.e., the structures of irregular shape, with a degree of irregularity increasing when the liquid lipid components used were of natural origin. Figure 7 shows the diffractograms recorded for the solid lipid (Imwitor^®^ 900 K) and the lipid nanoparticle dispersions. The crystallization profile of the solid lipid contains characteristic signals at the angles 2Θ = 20° and 23°. These typical diffraction reflexes were also observed on the diffractograms of the dispersions of the lipid nanoparticles studied. The ones observed for the dispersions were much less intense and slightly shifted towards lower values of angle 2Θ, which is, however, typical of lipid nanoparticles and confirms the presence of the polymorphic β′ form, characteristic of the lipids based on triacylglycerols in the colloidal state [40]. The XRD results were fully consistent with the results of DSC analysis (Figure 8). Briefly, Imwitor^®^ 900 K, as a triacylglycerol-based lipid, in the solid state is predominantly in the stable β form, is responsible for a single peak at about 65 °C in the thermogram. The conversion of the solid lipid to the colloidal state, associated with the formation of the lipid nanoparticles, resulted in a decrease in the melting point of the lipid nanoparticle dispersions [41] by about 3 °C for the NLCs with Miglyol^®^ 810 N and microalgae oil and noticeably less for the lipid extracted from diatoms. This observation confirmed the efficient incorporation of the tested liquid lipids into the lipid nanoparticle matrices, not affecting the polymorphic form and stability of the lipid matrix itself.

### 3.5. Evaluation of Stability of Investigated Lipid Nanoparticles

The final step was to evaluate the stability of the obtained nanoparticle dispersions. The study included an evaluation of the changes in the values of average particle size, the polydispersity index and the zeta potential of the dispersions, stored at three temperatures for a period of 30 days (Table 6). Thus, for the nanoparticles containing Miglyol^®^ 810 N as a liquid lipid, no significant changes in the parameter values were observed during the storage time. The only exception was the sample stored at a reduced temperature (4 °C), for which changes in the PDI value were noted. For the nanoparticles containing microalgae oil, the results were somewhat different. The mean particle size and PDI showed notable changes over time for the samples subjected to reduced and increased (40 °C) temperatures. The most favorable results were obtained for the dispersions stored at room temperature (25 °C) and this is the one recommended for storage of the obtained NLCs. Analogous results were obtained for nanoparticles enriched with *Halamphora* cf. *salinicola* (SZCZM1454A) crude oil; although, particle size fluctuations were not as pronounced as for the NLCs with microalgae oil alone. Room temperature was also recommended for storage.

Stability studies of nanostructured lipid carriers based on another natural lipid—Siberian pine seed oil—were conducted by a research group from the Siberian Branch of the Russian Academy of Sciences [42]. Their results indicated that the increasing amount of natural oil up to 5% does not lead to instability of NLC dispersions and their stability is independent of storage temperature. The reason can be the method used to produce lipid nanoparticles, which was hot pressure homogenization, which can favorably improve the stability of nanoparticles over time, but it requires the use of high pressure and a special homogenizer for the process. Additionally, the results reported by Yang and co-workers [43] indicate that the type and amount of liquid lipids significantly affect the stability of the lipid nanoparticles containing it.

## 4. Conclusions

The use of lipid components of diatoms in the production of biodegradable lipid nanoparticles seems to be highly justified from the point of view of zero-waste tendency assumed also in chemical and pharmaceutical industries. The advantages are also seen in the composition of beneficial fatty acids contained in *Halamphora* cf. *salinicola* (SZCZM1454A) crude oil, whose presence was confirmed by GC-MS analysis. Additionally, the preparation of stable lipid nanocarriers, enriched with microalgae oil (*Schizo-chytrium*) and lipids extracted from *Halamphora*, by applying the method which does not require high pressure or ultrasound, has been described for the first time. The outcome of our study was the production of NLCs with desirable physicochemical parameters of lipid nanoparticles for the pharmaceutical/cosmetic industry (particle size below 200 nm, PDI not exceeding 0.3, ZP higher than |±30 mV|).

## 5. Patents

The detailed procedure of the production of the nanostructured lipid nanocarriers mentioned in this work is covered by a patent application in Poland—no. P.438767 and an European patent application—no. EP22460021 (Marzec M, Nowak I, Dąbek P, Witkowski A. A method of obtaining of lipid nanoparticles synthesised on the basis of marine microalgae (*Schizochytrium*) and lipids obtained from diatoms (*Halamphora*)).

## Figures and Tables

**Table 1:** 3^3^ factorial design—the quantitative composition of the NLC dispersions—assumptions of the experiment.

	Quantity of Ingredient (wt.% ± 0.1)		
Sample No.	Solid Lipid	Liquid Lipid	Non-Ionic Surfactant
1	4.0	1.0	0.5
2	4.0	1.0	1.0
3	4.0	1.0	1.5
4	4.0	1.5	0.5
5	4.0	1.5	1.0
6	4.0	1.5	1.5
7	4.0	2.0	0.5
8	4.0	2.0	1.0
9	4.0	2.0	1.5
10	4.5	1.0	0.5
11	4.5	1.0	1.0
12	4.5	1.0	1.5
13	4.5	1.5	0.5
14	4.5	1.5	1.0
15	4.5	1.5	1.5
16	4.5	2.0	0.5
17	4.5	2.0	1.0
18	4.5	2.0	1.5
19	5.0	1.0	0.5
20	5.0	1.0	1.0
21	5.0	1.0	1.5
22	5.0	1.5	0.5
23	5.0	1.5	1.0
24	5.0	1.5	1.5
25	5.0	2.0	0.5
26	5.0	2.0	1.0
27	5.0	2.0	1.5

**Table 2:** 3^2^ factorial design—the synthesis condition of the NLC dispersions—assumptions of the experiment.

	Speed of the Two-Stage High Shear Homogenization (rpm)	
Sample No.	First Stage	Second Stage
1	24,000	13,500
2	24,000	9500
3	24,000	8000
4	20,500	13,500
5	20,500	9500
6	20,500	8000
7	9500	8000
8	13,500	8000

**Table 3:** Fatty acid profile in the crude oil sample.

Assigned FA ^1^	Name	RSD ^2^ (%)	TFA ^3^ (%)
C7:0	Heptanoic acid	15.03	0.01
C8:0	Octanoic acid	11.22	0.01
C8:1n-6	2-Octenoic acid	13.79	0.01
C9:0	Nonanoic acid	4.61	0.02
8-Me-C9:0	8-Methylnonanoic acid	14.41	0.04
C10:0	Decanoic acid	12.77	0.01
C11:0	Undecanoic acid	16.05	0.02
C12:0	Dodecanoic acid	8.54	0.27
C13:0	Tridecanoic acid	11.22	0.13
12-Me-C13:0	12-Methyltridecanoic acid	14.44	0.09
C14:0	Tetradecanoic acid	18.40	2.08
11-Me-C14:0	11-Methyltetradecanoic acid	16.61	15.92
13-Me-C14:1	13-Methyltetradec-9-enoic acid	19.15	0.15
4,8,12-Me-C13:0	4,8,12-Trimethyltridecanoic acid	15.35	0.09
13-Me-C14:0	13-Methyltetradecanoic acid	20.33	0.43
12-Me-C14:0	12-Methyltetradecanoic acid	16.33	0.35
C15:1n-5	10-Pentadecenoic acid	19.48	0.25
C15:0	Pentadecanoic acid	17.75	1.95
C16:1n-14	2-Hexadecenoic acid	16.75	0.04
C16:1n-7	9-Hexadecenoic acid	18.38	35.94
C16:1n-5	11-Hexadecenoic acid	16.11	0.92
C16:1n-9	7-Hexadecenoic acid	22.37	0.09
C16:0	Hexadecanoic acid	18.34	26.44
C16:2n-4,7	9,12-Hexadecadienoic acid	11.90	0.10
5,9,13-Me-C14:0	5,9,13-Trimethyltetradecanoic acid	17.94	1.18
C16:2n-6,9	7,10-Hexadecadienoic acid	19.32	0.17
15-Me-C16:0	15-Methylhexadecanoic acid	23.32	0.18
14-Me-C16:0	14-Methylhexadecanoic acid	22.80	0.30
C17:1n-9	8-Heptadecenoic acid	14.11	0.11
C17:1n-7	10-Heptadecenoic acid	16.17	0.10
C17:0	Heptadecanoic acid	13.90	0.23
C18:3n-6,9,12	6,9,12-Octadecatrienoic acid	14.10	0.13
C18:2n-6,9	9,12-Octadecadienoic acid	20.43	1.21
C18:1n-9	9-Octadecenoic acid	14.34	4.06
C18:1n-7	11-Octadecenoic acid	24.21	2.52
C18:0	Octadecanoic acid	17.20	0.75
11-Me-C18:1	11-Methyloctadec-12-enoic acid	20.48	1.01
10-Me-C18:0	10-Methyloctadecanoic acid	22.98	0.08
C19:1n-9	10-Nonadecenoic acid	17.34	0.40
C20:4n-6	5,8,11,14-Eicosatetraenoic acid	17.25	0.21
C20:5n-3	5,8,11,14,17-Eicosapentaenoic acid	17.35	0.40
C20:1n-9	11-Eicosenoic acid	19.31	0.09
C20:0	Eicosanoic acid	22.78	0.08
C22:0	Docosanoic acid	23.84	0.10
C24:1n-9	15-Tetracosenoic acid	23.42	0.07
C24:0	Tetracosanoic acid	17.83	1.28

^1^ FA = fatty acid; ^2^ RSD = relative standard deviation in terms of percentage; ^3^ TFA = fraction of total fatty acids.

**Table 4:** Results of lipid screening—observation of homogeneity of mixtures (solid lipids combined with selected liquid lipids at the ratio 2:1) over time.

Liquid Lipid	Solid Lipid	Solubility				
		15 min	30 min	1 h	24 h	72 h
Miglyol^®^ 810 N	Compritol^®^ 888 ATO	+	+	+	+	+
	Precirol^®^ ATO 5	-	-	-	-	-
	Imwitor^®^ 900 K	+	+	+	+	+
	Softisan^®^ 601	+	+	+	+	+
Microalgae oil (*Schizochytrium*)	Compritol^®^ 888 ATO	+	+	+	+	+
	Precirol^®^ ATO 5	-	-	-	-	-
	Imwitor^®^ 900 K	+	+	+	+	+
	Softisan^®^ 601	+	+	+	-	-
*Halamphora* cf. *salinicola* crude oil with microalgae oil	Compritol^®^ 888 ATO	-	-	-	-	-
	Precirol^®^ ATO 5	-	-	-	-	-
	Imwitor^®^ 900 K	+	+	+	+	+
	Softisan^®^ 601	+	+	+	-	-

“+” soluble; “-” insoluble.

**Table 5:** 3^3^ factorial design—response values (Z-Ave, PDI and ZP) of the independent factors of 27 experiments for lipid nanoparticles containing each liquid lipids tested (data are expressed as mean ± standard deviation).

	Miglyol^®^ 810 N			Microalgae Oil (*Schizochytrium*)			*Halamphora* cf. *salinicola* Crude Oil with Microalgae Oil		
Sample No.	Z-Ave ^1^ (nm)	PDI ^2^ (-)	ZP ^3^ (mV)	Z-Ave ^1^ (nm)	PDI ^2^ (-)	ZP ^3^ (mV)	Z-Ave ^1^ (nm)	PDI ^2^ (-)	ZP ^3^ (mV)
1	232.6 ± 5.9	0.166 ± 0.016	53.0 ± 1.4	236.1 ± 1.0	0.158 ± 0.031	51.4 ± 1.4	220.3 ± 3.6	0.193 ± 0.011	50.8 ± 1.3
2	186.8 ± 5.3	0.198 ± 0.018	52.6 ± 0.2	198.8 ± 6.8	0.172 ± 0.019	49.4 ± 2.4	218.9 ± 2.8	0.224 ± 0.011	50.7 ± 1.3
3	145.8 ± 1.6	0.232 ± 0.012	51.1 ± 1.0	178.1 ± 3.0	0.195 ± 0.012	50.7 ± 1.8	211.5 ± 6.5	0.250 ± 0.010	49.5 ± 0.6
4	201.1 ± 4.7	0.155 ± 0.024	51.0 ± 1.3	223.0 ± 4.9	0.185 ± 0.010	50.8 ± 0.6	204.1 ± 0.7	0.181 ± 0.018	50.8 ± 1.1
5	205.2 ± 2.6	0.159 ± 0.013	50.5 ± 0.5	215.3 ± 2.8	0.184 ± 0.008	49.0 ± 1.5	226.0 ± 2.2	0.264 ± 0.013	49.6 ± 1.9
6	155.4 ± 1.8	0.160 ± 0.012	49.1 ± 2.1	173.9 ± 1.3	0.149 ± 0.009	48.9 ± 0.4	177.3 ± 2.9	0.180 ± 0.005	49.4 ± 0.6
7	190.1 ± 2.2	0.119 ± 0.004	50.6 ± 1.0	239.3 ± 5.3	0.163 ± 0.013	51.6 ± 0.1	246.2 ± 4.2	0.194 ± 0.013	53.3 ± 1.8
8	195.0 ± 2.6	0.155 ± 0.007	49.4 ± 0.5	238.2 ± 5.2	0.161 ± 0.018	43.4 ± 1.6	217.9 ± 2.5	0.141 ± 0.019	50.5 ± 0.9
9	151.8 ± 1.7	0.152 ± 0.018	51.5 ± 3.4	199.5 ±3.4	0.158 ± 0.019	46.4 ± 0.8	237.4 ± 4.3	0.247 ± 0.004	50.3 ± 1.4
10	196.7 ± 6.6	0.175 ± 0.006	48.3 ± 1.2	219.6 ±3.5	0.162 ± 0.002	47.5 ± 1.3	194.4 ± 1.3	0.149 ±0.010	53.2 ± 2.2
11	203.6 ± 4.2	0.186 ± 0.010	51.4 ± 0.7	175.5 ± 2.4	0.146 ± 0.010	49.7 ± 0.6	163.1 ± 2.8	0.134 ± 0.016	50.4 ± 0.9
12	156.9 ± 3.0	0.218 ± 0.006	49.7 ± 1.0	170.3 ± 3.3	0.190 ± 0.015	50.0 ± 1.2	172.8 ± 1.7	0.190 ± 0.005	50.5 ± 1.2
13	241.8 ± 5.4	0.160 ± 0.020	51.5 ± 1.3	249.0 ± 3.3	0.169 ± 0.014	49.0 ± 0.9	253.4 ± 1.9	0.187 ± 0.004	44.5 ± 0.5
14	194.5 ± 3.5	0.194 ± 0.014	50.1 ± 0.8	190.0 ± 2.7	0.152 ± 0.012	51.3 ± 1.2	230.2 ± 4.4	0.195 ± 0.008	52.9 ± 1.2
15	179.9 ± 3.2	0.178 ± 0.018	49.9 ± 0.6	171.2 ± 1.6	0.163 ± 0.019	48.3 ± 1.4	175.9 ± 0.4	0.158 ± 0.006	45.9 ± 1.1
16	216.6 ± 3.1	0.122 ± 0.021	52.7 ± 0.6	253.9 ± 3.4	0.134 ± 0.025	51.1 ± 0.6	252.0 ± 0.9	0.172 ± 0.033	50.2 ± 0.4
17	207.2 ± 5.3	0.157 ± 0.010	49.4 ± 1.3	238.3 ± 5.1	0.162 ± 0.031	47.7 ± 1.1	228.9 ± 3.1	0.198 ± 0.010	51.4 ± 1.2
18	179.9 ± 3.1	0.147 ± 0.005	51.2 ± 1.9	171.9 ± 3.3	0.144 ± 0.012	45.7 ± 0.8	248.2 ± 3.9	0.224 ± 0.018	51.3 ± 1.1
19	280.8 ± 6.9	0.180 ± 0.018	53.9 ± 2.3	225.1 ± 5.6	0.135 ± 0.005	50.2 ± 0.7	259.7 ± 5.3	0.209 ± 0.013	49.7 ± 1.7
20	247.4 ± 7.4	0.259 ± 0.006	48.4 ± 1.3	178.0 ± 3.3	0.158 ± 0.015	45.1 ± 3.7	203.0 ± 3.7	0.178 ± 0.009	51.7 ± 0.6
21	209.3 ± 4.4	0.234 ± 0.006	48.7 ± 1.7	180.9 ± 4.1	0.181 ± 0.020	48.8 ± 0.8	203.9 ± 3.6	0.210 ± 0.025	48.9 ± 0.3
22	253.1 ± 9.0	0.129 ± 0.018	53.1 ± 0.6	249.5 ± 2.7	0.157 ± 0.012	51.3 ± 0.8	241.4 ± 4.1	0.150 ± 0.017	49.9 ± 1.6
23	195.2 ± 3.4	0.155 ± 0.015	50.8 ± 0.5	202.0 ± 1.9	0.153 ± 0.016	50.3 ± 0.8	189.9 ± 1.4	0.175 ± 0.010	43.4 ± 0.1
24	189.5 ± 4.3	0.210 ± 0.009	47.9 ± 1.8	174.2 ± 5.2	0.183 ± 0.018	47.3 ± 2.6	211.6 ± 1.7	0.195 ± 0.023	46.8 ± 0.6
25	245.7 ± 3.3	0.124 ± 0.021	53.1 ± 1.4	263.7 ± 5.8	0.137 ± 0.041	51.7 ± 0.5	272.2 ± 4.7	0.158 ± 0.016	49.6 ± 1.5
26	215.4 ± 4.3	0.178 ± 0.005	48.5 ± 1.0	225.0 ± 2.1	0.149 ± 0.024	47.8 ± 3.0	209.5 ± 3.1	0.178 ± 0.014	49.0 ± 2.3
27	173.1 ± 3.0	0.163 ± 0.011	49.9 ± 1.6	194.2 ± 3.4	0.144 ± 0.010	44.1 ± 1.2	190.6 ± 2.3	0.170 ± 0.031	51.4 ± 1.3

^1^ Z-Ave = mean particle size; ^2^ PDI = polydispersity index; ^3^ ZP = zeta potential (pH of solutions within the range 6.2–6.5); RI = refractive index = 1.45.

**Table 6:** Stability study—changes in Z-Ave, PDI and ZP of lipid nanoparticles stored in various temperature conditions (4, 25, and 40 °C) for 30 days (data are expressed as mean ± standard deviation).

Temp.	Day	Miglyol^®^ 810 N	Microalgae Oil (*Schizochytrium*)	*Halamphora* cf. *salinicola* Crude Oil with Microalgae Oil
		**Mean particle Size (nm)**		
**25 °C**	**1**	170.1 ± 0.7	184.2 ± 0.8	216.4 ± 1.5
**4 °C**	**30**	192.6 ± 11.6	465.5 ± 8.7	301.3 ± 9.6
**25 °C**	**30**	168.2 ± 1.4	168.0 ± 2.1	206.9 ± 2.3
**40 °C**	**30**	174.5 ± 1.2	486.6 ± 8.4	245.3 ± 9.2
		**Polydispersity Index (-)**		
**25 °C**	**1**	0.196 ± 0.014	0.189 ± 0.002	0.198 ± 0.012
**4 °C**	**30**	0.293 ± 0.024	0.508 ± 0.055	0.450 ± 0.020
**25 °C**	**30**	0.168 ± 0.012	0.150 ± 0.009	0.175 ± 0.003
**40 °C**	**30**	0.192 ± 0.001	0.556 ± 0.073	0.403 ± 0.044
		**Zeta Potential (mV) ^1^**		
**25 °C**	**1**	48.3 ± 1.6	49.1 ± 0.6	50.6 ± 1.1
**4 °C**	**30**	49.9 ± 1.0	53.6 ± 0.7	50.0 ± 1.8
**25 °C**	**30**	52.0 ± 0.7	50.1 ± 0.7	51.1 ± 0.2
**40 °C**	**30**	51.7 ± 0.4	47.2 ± 0.2	54.1 ± 2.1

^1^ pH of solutions within the range 6.2–6.5.

## Data Availability

The data presented in this study are available upon request from the authors.
